# Antibacterial and Antifungal Properties of Polyester, Polylactide, and Cotton Nonwovens and Fabrics, by Means of Stable Aqueous Dispersions Containing Copper Silicate and Some Metal Oxides

**DOI:** 10.3390/ma16165647

**Published:** 2023-08-16

**Authors:** Jerzy J. Chruściel, Joanna Olczyk, Marcin H. Kudzin, Piotr Kaczmarek, Paulina Król, Nina Tarzyńska

**Affiliations:** 1 Łukasiewicz Research Network—Lodz Institute of Technology, Brzezińska 5/15, 92-103 Łódź, Poland; joanna.olczyk@lit.lukasiewicz.gov.pl (J.O.); marcin.kudzin@lit.lukasiewicz.gov.pl (M.H.K.); piotr.kaczmarek@lit.lukasiewicz.gov.pl (P.K.); paulina.krol@lit.lukasiewicz.gov.pl (P.K.); nina.tarzynska@lit.lukasiewicz.gov.pl (N.T.); 2 Circular Economy Center (BCG), Environmental Protection Engineering Research Group, Brzezińska 5/15, 92-103 Łódź, Poland; 3 Biodegradation and Microbiological Research Laboratory, Brzezińska 5/15, 92-103 Łódź, Poland; 4 Biomedical Engineering Center, Marii Skłodowskiej-Curie 19/27, 90-570 Łódź, Poland

**Keywords:** antimicrobial modification of textile materials, antibacterial and antifungal properties, biofunctionalization, polyester, poly (lactic acid) (PLA), nonwovens, PES and cotton fabrics, copper silicate hydrate, CuSiO_3_, titanium dioxide, TiO_2_, zinc oxide, hybrid composite oxide ZnO∙SiO_2_, air filters suitable for air-conditioning apparatus

## Abstract

Literature reviews have described the applications of silver, copper, and zinc ions and metallic particles of Cu, Ti, and Zn oxides, which have been found to be useful antimicrobial reagents for the biofunctionalization of various materials and their surfaces. For this purpose, compositions of water dispersions containing emulsions of synthetic copolymers based on acrylic and vinyl monomers, polysaccharides (hydroxyethyl cellulose and starch), and various additives with wetting and stabilizing properties were used. Many stable water dispersions of different chemical compositions containing bioactive chemical compounds (copper silicate hydrate, titanium dioxide, and zinc oxide (and other auxiliary substances)) were developed. They were used for the preparation of thin hybrid coatings having good antimicrobial properties against Gram-negative bacteria (*Escherichia coli*), Gram-positive bacteria (*Staphylococcus aureus*), and yeast fungus (*Candida albicans*). Polyester (PES) and polylactide (PLA) nonwovens were modified using the dip-coating method, while PES and cotton fabrics were biofunctionalized by means of dip-coating and coating methods. The antimicrobial (antibacterial and antifungal) properties of the textile materials (nonwovens and fabrics) biofunctionalized with the above-mentioned bioactive agents exhibiting antimicrobial properties (CuSiO_3_, TiO_2_, ZnO, or ZnO∙SiO_2_) were strongly dependent on the agents’ content in the water dispersions. The PES and PLA nonwovens, modified on the surface with water compositions containing copper silicate hydrate, showed good antibacterial properties against the Gram-negative bacteria *Escherichia coli*, even at a content of 1 wt.% CuSiO_3_∙xH_2_O, and against the Gram-positive bacteria *Staphylococcus aureus*, at the content of at least 5 wt.% CuSiO_3_∙xH_2_O. The bacterial growth reduction factor (R) was greater than 99% for most of the samples tested. Good antifungal properties against the fungus *Candida albicans* were found for the PES and PLA nonwoven fabrics modified with dispersions containing 5–7 wt.% CuSiO_3_∙xH_2_O and 4.2–5.0 wt.% TiO_2_. The addition of TiO_2_ led to a significant improvement in the antifungal properties of the PES and PLA nonwovens modified in this way. For the samples of PES *WIFP-270* and *FS F-5* nonwovens, modified with water dispersions containing 5.0 wt.% CuSiO_3_∙xH_2_O and 4.2–5.0 wt.% TiO_2_, the growth reduction factor for the fungus *Candida albicans* (R) reached values in the range of 80.9–98.0%. These new biofunctionalized polymeric nonwoven textile materials can find practical applications in the manufacture of filters for hospital air-conditioning systems and for the automotive industry, as well as in air purification devices. Moreover, similar antimicrobial modification of fabrics with the dip-coating or coating methods can be applied, for example, in the fabrication of fungi- and mold-resistant garden furniture.

## 1. Introduction

In the literature, for a long time, the properties of silver [1] and zinc [2] ions, as well as copper microparticles (MPs), nanoparticles (NPs), and their surfaces [3,4], have been known, showing a strong inhibitory effect on the growth of different bacteria. The antimicrobial properties of silver MPs and NPs have great application potential in the textile industry and in medicine [5,6,7,8,9,10,11,12]. Silica nanofibers containing Ag NPs [13] and PLA–chitosan composite fibers [14] also exhibit excellent antibacterial properties.

Copper (II) oxide (CuO) also has antibacterial, antifungal, and even antiviral properties [15,16,17,18,19,20]. Although for most microorganisms, low concentrations of copper are sufficient, usually much higher doses of Cu (even in the amount of 3–10 wt.%) have been used to inhibit the growth of some microorganisms and provide antimicrobial features [21,22]. Research on modifying the properties of polymeric and textile materials in order to obtain more effective and economical methods of antimicrobial protection has been ongoing in the recent two decades. A constant development of research on the antibacterial properties of materials containing Cu NPs has been observed [4,22,23,24,25,26].

From the publication of C.C. Trapalis et al. [27], the method of obtaining thin composite silicate coatings containing copper particles (CuPs) deposited on glass plates using a sol–gel method (Cu/silica (SiO_2_)) is known. The fabricated coatings showed high antibacterial activity against *Escherichia coli* bacteria, which increased with the increase in the metal content and decreased with the increase in the thermal treatment temperature in a deposition process of metal nanoparticles on different surfaces. The CuPs incorporated onto nano-silica also showed an inhibitory effect on the growth of microorganisms. They were also used to remove the odor of mercaptans [28]. The CuPs immobilized onto the surface of SiO_2_ spheres did not aggregate and showed good antimicrobial properties against colonies of *Escherichia coli*, *Staphylococcus aureus*, and *Candida albicans* when the concentration of SiO_2_@Cu was higher than 500 µg/mL [29]. Silica NPs with a “core–shell” structure (nucleus–shell), containing approximately 0.1 μg of Cu (in the form of insoluble copper hydroxide), had much better antibacterial properties against *Escherichia coli* and *Bacillus subtilis* than those observed with Cu(OH)_2_ alone, and the *minimum inhibitory concentration* (MIC) of these bacteria was 2.4 μg Cu/mL in the case of SiO_2_@Cu having the “core–shell” structure [30].

Thin layers of Cu–SiO_2_ nanocomposites (NCs) obtained with chemical vapor deposition (CVD) showed good antibacterial properties against numerous pathogens found in hospital conditions (*Acinetobacter baumannii*, *Klebsiella pneumoniae*, *Stenotrophomonas maltophilia*, *Enterococcus faecium*, *Staphylococcus aureus*, and *Pseudomonas aeruginosa*). These coatings with microbiological properties can be used for protection of metal and ceramic surfaces [31]. It has also been observed that copper and zinc alginates and their composites with silica have a stronger antibacterial effect against *Enterococcus faecalis* bacteria than in the case of ordinary solutions of Cu and Zn salts [32].

Cu NPs deposited on the surface of sodium aluminosilicate (sodium montmorillonite, MMT) or intercalated inside its layered structure showed high stability in an air atmosphere (over 3 months) and excellent microbiological activity against various colonies of bacteria (*Escherichia coli* [33], *Staphylococcus aureus*, *Pseudomonas aeruginosa*, and *Enterococcus faecalis*), causing the disappearance of >90% of bacteria after 12 h. Cytotoxicity studies have shown a minimal adverse effect of this nanocomposite (NC) on human cells when the MIC of these microorganisms is exceeded. Nevertheless, promising prospects for the use of the MMT–Cu NC for therapeutic purposes are anticipated [4]. It was reported that other metallic and metal oxide NPs also exhibit antimicrobial properties [34]. Monodisperse copper nanoparticles (with dimensions 2–5 nm), deposited on magnesium silicate [Mg_8_Si_12_O_30_(OH)_4_(H_2_O)_4_·8H_2_O] (sepiolite), had strong antibacterial properties (against *Staphylococcus aureus* and *Escherichia coli*) which turned out to be comparable to the biological activity of *Triclosan* [35]. Spherical NCs of copper and silver with mullite (3Al_2_O_3_·2SiO_2_) also showed a strong antibacterial effect against *Escherichia coli* and *Staphylococcus aureus*. The microbiological activity of the Cu-mullite NC turned out to be higher than that of the mullite-Ag NC, presumably due to the smaller particle size of this composite. Both NCs showed good cytocompatibility at a concentration of 1 mg/mL (MBC) and therapeutic properties in the treatment of wounds in mice [36].

A suspension of an emulsion paint with the addition of a mixture of 0.1–20 wt.% anhydrous copper silicate (CuSiO_3_) with vegetable oils was used to coat the inner surfaces of pots and containers used for growing plants and flowers [37]. In the sol–gel process, mesoporous copper silicate xerogels with a high specific surface area (463 m^2^/g) and a pore size of 2 nm were obtained, showing antibacterial properties that were dependent on the CuP concentration [38].

T. Jesionowski et al. prepared polyester (PES) films containing a 2 or 8 wt.% hybrid oxide composite CuO∙SiO_2_, which exhibited excellent antibacterial properties against *Pseudomonas aeruginosa* bacteria [39]. Similar composites of polyolefins (LDPE or PP) containing 2–8 wt.% CuO∙SiO_2_ had increased thermal resistance and thermal diffusivity as well as good biocidal properties [40]. Silica NCs containing Cu (0) MPs and Cu(I) compounds showed higher antibacterial effectiveness than Cu (II) compounds—against *Xanthomonas alfalfae* and *Escherichia coli* bacteria. A phytotoxicity study under greenhouse conditions (with the participation of *Vinca* sp. and *Hamlin orange*) provided NCs that were safe for plants and could be used as biocides in agriculture [41].

In the previous years, many research works conducted in our institute have been concerned with the incorporation of metal oxides into the structure of textile materials, providing good antimicrobial properties. It was found that micronized particles of metal oxides (ZnO, TiO_2_), introduced into the structure of a polyester nonwoven fabric with the dip-coating procedure, showed bioactivity against various bacterial colonies and selected fungi, as well as properties of absorption of UV light and the ability to photo-oxidize organic substances. Textile materials modified in this way could find many practical applications, especially in the production of protective clothing [42,43,44]. In addition, a polyester nonwoven fabric and a cotton fabric, modified with dispersions containing ZnO or the ZnO·SiO_2_ composite of 30 wt.% ZnO content, showed barrier properties against UV radiation, as expressed by the ultraviolet protection factor (UPF) > 45 and high absorption of UV radiation in the entire spectral range [45,46].

In the following years, a method of antimicrobial functionalization of polypropylene (PP) and polylactide (PLA) nonwovens, applying the melt-blown extrusion process, was developed using copper silicate hydrate (CuSiO_3_∙xH_2_O), with the addition of a wide range of various plasticizers (most often PEG600). The obtained PP and PLA nonwoven fabrics had excellent antibacterial properties against Gram-negative bacteria *Escherichia coli* and against Gram-positive bacteria *Staphylococcus aureus* and also good antifungal properties against the *Candida albicans* yeast fungus, even at a quite low CuSiO_3_ hydrate content in these fiber composites of 0.5–1 wt.%. The growth reduction factor for these microorganisms (R) was greater than 98% [47,48,49,50]. Analogously, using the melt-blown technique, composite nonwoven fabrics were obtained from mixtures of polymers (PP and PLA, 1:1) with 0.5 wt.% copper silicate hydrate (CuSiO_3_∙xH_2_O, containing 18.5% of H_2_O), with the addition of 5 wt.% paraffin oil as the plasticizer. The PLA/PP/paraffin/CuSiO_3_ composite nonwoven fabric had strong antibacterial properties as well—against *Escherichia coli* and *Staphylococcus aureus* bacteria. Thermal properties of all types of these composite nonwovens were tested using the DSC method. Physicochemical parameters, the specific surface area, surface morphology (with the SEM method), the element content (with the EDS method), and resistance to hydrolytic degradation (in alkaline and neutral environments) were also determined [51].

Due to the constant increase in the consumption of materials made of biodegradable polymers on a global scale, research on the biofunctionalization of composite nonwovens with both conventional (PP and PES) and biodegradable polymers is of great importance, among which poly (lactic acid) (PLA) is the most important and widely used. The wide use of PLA results from its availability, good mechanical properties, and relative ease of processing with various techniques (e.g., the melt-blown technique) [52,53]. PLA has been used, among others, in medicine, in the production of various types of packaging that come into contact with food, and for obtaining a new generation of textile materials [53,54].

Biodegradable polymers are more expensive than traditional polymers and have slightly worse mechanical properties, as some of them in the pure state are, for example, quite stiff or brittle [52]. For this reason, in their processing, various plasticizers are used as additives. Poly (ethylene glycol) (PEG) is the most commonly used plasticizer for PLA [55] and polyhydroxyalkanoates (PHA) [53]. It was found that the addition of PEG200 to PLA in an amount of 1–7 wt.% causes a gradual decrease in the glass transition temperature (T_g_) of PLA–PEG200 mixtures from 62.9 °C to 48.5 °C, and at a content of 10 wt.%, PEG200 T_g_ reached 51.6 °C [55]. In contrast, the flexibility and hydrophilicity of coatings made of poly(3-hydroxybutanoate) (P(3HB)) with PEG was greater than in the case of coatings made of P(3HB) alone, leading to the increased biocompatibility of such composite biomaterials [56].

Linen fabrics modified with 5–7 wt.% copper silicate with the dip-coating method showed excellent antimicrobial properties and barrier properties against UV radiation (with a high value of the UPF coefficient of 64–131 and a UV light transmittance of 0.20–3.40) [57]. Also, nonwoven PLA composites with copper alginate exhibited good antibacterial properties (against *Escherichia coli* and *Staphylococcus aureus*), antifungal activity (against *Aspergillus niger* and *Chaetomium globosum*), and barrier properties against UV radiation (UPF > 40) [58].

Fabrics modified on the surface with CuO also had good antibacterial properties and were non-toxic to the human skin. Thus, they can be used for the production of medical clothing [59]. Moreover, CuO nanoparticles showed a significant inhibitory effect on the development of *hepatitis C virus* (HCV) and effectively inactivated many other viruses, such as *rhinovirus 2*, *yellow fever virus*, *human influenza A*, *measles* and *parainfluenza type 3*, *Punta Toro*, *adenovirus type 1*, *cytomegalovirus*, *vaccinia*, and *herpes simplex type 1*. This proved the positive effect of CuO NPs on the growth of the human body’s immunity. Recently, the disappearance of SARS-CoV-2 from copper surfaces after 4 h was observed [60,61]. In contrast, polyurethane coatings containing Cu_2_O, applied to glass or stainless steel, quickly inactivated SARS-CoV-2 (99.9% within 1 h) [62]. The copper particles (CuPs) also inactivated several infectious viruses: *bronchitis virus*, *polio virus*, *human immunodeficiency virus type 1* (*HIV-1*), other enveloped and non-enveloped viruses, and single- and double-stranded DNA and RNA viruses. Thus, a strong contact-killing ability of copper surfaces against several viruses, including SARS-CoV-2, was confirmed. The increase in the copper content in plasma may enhance both innate and adaptive immunity in humans. Due to their strong antiviral activity, CuPs may also have preventive and therapeutic effects against COVID-19 [63].

Recently, cotton fabrics modified with Ag, ZnO, and Ag/ZnO NPs were developed, and their bactericidal activity against *Escherichia coli* and *Staphylococcus aureus*, photocatalytic activity, and antiviral activity against Delta SARS-CoV-2 were tested. These fabrics showed antimicrobial properties against both bacteria, but they reduced only part of the SARS-CoV-2 virions during the first 15 min of direct contact and caused damage only to the biological structures on the viral surface particle, while the viral RNA remained intact [64]. 

Alternatively, a cotton fabric functionalized with kaolinite-titania nano-hybrid particles enhanced the photoactivity of the immobilized TiO_2_ and led to the complete disinfection of *Escherichia coli* (Gram-negative) and *Bacillus cereus* (Gram-positive) bacteria during contact with this fabric [65]. Cotton fibers treated in situ with ammonium-salicylidene chitosan Schiff base, TiO_2_, and ZnO NPs exhibited strong antimicrobial activity against *S. aureus*, *E. coli*, and *C. albicans* pathogens and barrier properties toward UV radiation. The ZnO-rich nanocomposite endowed cotton fabrics with more UV protection than the TiO_2_-rich nanocomposite [66].

Copper silicate is a non-toxic reagent, and all antimicrobial agents, which were used in this study, are much cheaper than silver microparticles and their compounds. Thus, they should find many different practical applications soon, especially in the development of a technology for the production of a new group of textile materials with strong antimicrobial (antibacterial and antifungal) properties and even for deactivating various viruses, e.g., SARS-CoV-2.

## 2. Experimental Part

### 2.1. Materials

Polyester nonwovens:

- WIFP-270 with a surface mass of approx. 270 g/m^2^ (Łukasiewicz Research Network–Lodz Institute of Technology, Łódź, Poland),
- Filter nonwovens: *FS G-4* with a surface mass of approx. 210 g/m^2^ and *FS F-5* with a surface mass of approx. 240 g/m^2^ (Filter Service Ltd., Zgierz, Poland),
- Polyester nonwoven (*aqua-jet*, *Hydronina*) having a surface mass of approx. 100 g/m^2^ (Lentex, Lubliniec, Polska),
- Polylactide nonwoven fabric (*PLA-350*) with a surface mass of approx. 350 g/m^2^ (ZPHU Gramix, Brzeziny, Poland),
- Polyester fabric with a twill weave and a surface mass of approx. 140 g/m^2^ (Andropol S.A., Andrychów, Poland).

Textile fabrics:

- Cotton–polyester fabric with 70 wt.% content of PES (*Figaro*), having a twill weave and a surface mass of approx. 170 g/m^2^ (Andropol S.A., Andrychów, Poland),
- Cotton fabric (*Medical*) with a plain weave and a surface mass of approx. 150 g/m^2^ (Andropol S.A., Andrychów, Poland).

### 2.2. Chemical Reagents

List of chemical reagents:

1. Copper sulfate CuSO_4_∙5H_2_O, pure (Chempur, Piekary Śląskie, Poland),
2. *Vitrosilicon 137S*, a water solution of sodium water glass, having a SiO_2_:Na_2_O molar ratio of 3.3 (CIECH Vitrosilicon, 68-120 Iłowa, Poland),
3. *CELLOSIZE HEC QP-40* (hydroxyethyl cellulose + sodium acetate) (Amerchol, Edison, NJ, USA)—a thickening agent,
4. Poly (ethylene glycol) *Polikol 400* (PEG400) (PCC Exol, Brzeg Dolny, Poland)—a wetting agent,
5. Poly (ethylene glycol) *Pluriol E600* (PEG600) (BASF, Ludwigshafen, Germany)—a wetting agent,
6. Water dispersion of styrene–acrylic resin *Revacryl 247*^®^ (Thorex, Łódź, Poland)—a binder.
7. Water dispersion of acrylic resin *Talens Amsterdam Acrylic Binder 005* (Talens, Amsterdam, Niderlands),
8. *Cinkarna CCA 100 BS*, a water dispersion of acrylic resin, containing 20–22 wt.% nano-TiO_2_ (~10 nm) (Cinkarna Celje, d.d., Slovenia),
9. Water dispersion of acrylic resin *Dekoral Silver* (PPG DECO Sp. z o.o., Wrocław, Poland)—a binder,
10. Acrylic photocatalytic water dispersion *Titanium IN* (Pigment, Szczecin, Poland)—a binder,
11. Silicate photocatalytic water dispersion *Titanium FA* (Pigment, Szczecin, Poland)—a binder,
12. *Synexil DN-50*, water dispersion of poly (vinyl acetate), PVAc (*Synthos S.A*., Oświęcim, Poland)—a binder,
13. Poly (vinyl alcohol) *Mowiol 4-98* (Fluka, Germany) with an average molecular weight (M_w_) of ~27,000 g/mol) — a thickening and pro-adhesion agent,
14. Soluble starch (Chempur, Piekary Śląskie, Poland)—a thickening and pro-adhesion agent,
15. Glycerin, pure (POCh, Gliwice, Poland)—a plasticizer,
16. Bis (2-etylohexyl adipate) (*Adoflex*) (Zakłady Azotowe Kędzierzyn S.A., Kędzierzyn-Koźle, Poland)—a plasticizer,
17. Nanosilica *Aerosil 380* (Evonik, Essen, Germany) — a stabilizer of dispersions,
18. Methyl silicone oil containing OH terminal groups, with a dynamic viscosity of 500 cP, *Polastosil M-200* (Zakład Chemiczny “*Silikony Polskie”*, Nowa Sarzyna, Poland)—an antifoaming agent,
19. Enzyme *Texazym PES* (INOTEX, Dvůr Králové n.L, Czechia);
20. Synthetic acrylic thickening agent *Lutexal Thickener HC* (BASF, Ludwigshafen, Germany)—for our purpose, diluted with demineralized water (1:3, *w*/*w*),
21. Copper silicate hydrate CuSiO_3_∙18.5H_2_O (Poznań University of Technology, Poznań, Poland) composed of 35.23 wt.% CuO, 62.16 wt.% SiO_2_, 18.52 wt.% H_2_O, 0.02 wt.% Na_2_O, and 0.01 wt.% K_2_O, with a particle diameter in the range of 1–100 µm [39],
22. Titanium dioxide, TiO_2_ (*TK44*) (Poznań University of Technology, Poznań, Poland), with an average particle diameter of 615 nm and a polydispersity of 0.102, obtained from the anatase allotrope of TiO_2_, having the commercial name *Tytanpol* (Police S.A., Szczecin, Poland), with an average particle diameter of 712–825 nm and a polydispersity of 0.218—modified with 1 wt.% N-2-aminoethyl-3-aminopropyl-(trimethoxy)silane,
23. Zinc oxide, ZnO (*Z11*) (Poznań University of Technology, Poznań, Poland), with an average particle diameter of 396 nm and a polydispersity of 0.161,
24. Zinc lactate, p.a. (Xenon-Chemists’ Cooperative, Rąbień, Poland),
25. Hybrid oxide ZnO∙SiO_2_, prepared as described in [46].

### 2.3. Modification of the Surface of Nonwovens and Polyester Fabrics before the Dip-Coating Process

The appropriate surface modification of textile materials, in particular hydrophobic polyester fiber, has a significant impact on the effectiveness of the process of biocide modifiers’ incorporation. In order to improve the wettability of the tested polymer nonwovens and increase the adhesion of the dispersion components to the surface of the nonwovens, the nonwovens were alkalized at room temperature by immersing them in a solution containing 2 wt.% NaOH and 4 wt.% Na_2_CO_3_ for 4 days. Next, the samples of nonwoven fabrics were drained of excess alkali solution in a horizontal two-shaft pad machine, immersed three times in demineralized water for 15 min, again drained of excess liquid in a horizontal double-shaft pad machine, then dried at 120 °C for 4 min, and heated at 140 °C for 2 min in the KTF-350S apparatus (Mathis, Oberhasli, Switzerland).

However, in order to increase wettability and improve adhesive properties, two methods of initial surface modification of the polyester fabric were used: (1) enzymatic treatment or (2) alkali treatment.

(1) Enzyme treatment was carried out at 35 °C for 30 min in a bath containing 1–2 wt.% enzyme from the group of esterases, Texazym PES, at pH 4.2 (adjusted with the addition of acetic acid). The bath ratio against polyester nonwovens was 10:1.
(2) Alkaline treatment was carried out at 98 °C for 60 min in a bath containing sodium hydroxide at a concentration of 1.8 g/L, sodium carbonate (3.6 g/L), and a sequestering and wetting agent. The bath ratio was 10:1.

#### 2.3.1. Synthesis of Copper Silicate Hydrate In Situ

Copper silicate hydrate (CuSiO_3_∙xH_2_O) was prepared, immediately before the dip-coating process of nonwoven fabrics, from diluted 5 wt.% solutions of copper (II) sulphate pentahydrate CuSO_4_∙5H_2_O) and sodium water glass (Na_2_SiO_3_) (*Vitrosilicon S-137S* with a silicate module: SiO_2_:Na_2_O = 3.32). However, for the preparation of aqueous dispersions containing more than 3 wt.% CuSiO_3_∙xH_2_O, the powdered copper silicate hydrate CuSiO_3_∙18.5H_2_O was used in this study — it was synthesized from copper nitrate and sodium water glass, as described in the literature [39].

#### 2.3.2. Description of the Dip-Coating Method for Polymer Nonwovens and Fabrics

Polymer nonwovens and fabrics were dip-coated with water dispersions containing copper silicate hydrate and other additives. Directly before the surfacing process, various auxiliary substances were added to the water dispersion of copper silicate: a wetting agent poly(ethylene glycol): *Polikol 400* or *Pluriol E600*), a thickening agent (e.g., hydroxyethyl cellulose, HEC), other modifiers, a dispersion stabilizer (nanosilica), and an antifoaming agent (methyl silicone oil) — in quantities adjusted to the assumed individual recipes of the surfacing process.

Most often, all aqueous dispersions (in demineralized water) contained 1.0 wt.% thickener based on hydroxyethyl cellulose (HEC) and generally 5–10 wt.% poly(ethylene glycol) (PEG) *Polikol 400* or *Pluriol E600*. In order to improve the stability of the water dispersions and their binding properties with respect to textile materials, various acrylic resin dispersions (e.g., *Revacryl 247*, *Talens Amsterdam Acrylic Binder 005*) and 2 wt.% aqueous solutions of starch or poly(vinyl alcohol) (PVA) were also added, and also, a small addition of *Aerosil 380* nanosilica (*Aerosil 380*) was used for most of the dispersions. Moreover, 0.1 or 0.2 wt.% antifoaming methyl silicone oil was added. When using a 2–4 wt.% water dispersion of poly (vinyl acetate) (PVAc), 1–2 wt.% bis (2-ethylhexyl adipate) (Adoflex) was added, and if a diluted starch solution was used, 0.4–1.0 wt.% glycerin was added—as a plasticizer.

Water dispersions containing copper silicate and auxiliary substances were used for the antimicrobial modification of polymer nonwovens. Some of the dispersions also contained TiO_2_, and one of the dispersions contained 5 wt.% CuSiO_3_ and zinc lactate. The chemical composition of the water dispersions was slightly modified during the tests, e.g., in the case of a higher content of CuSiO_3_∙xH_2_O (5–7% by weight), higher amounts of PEG400 or PEG600 (7.5–10% by weight) were used, as well as various binders (acrylic or other).

The aqueous dispersions were prepared by mixing the weighed ingredients in the amounts given in Table 1, using a four-blade *IKA RW 20* digital mechanical stirrer at a rotational speed of 2000 rpm for 2 min, and then, they were homogenized at a rotational speed of 20,000 rpm for 60 s using the digital homogenizer *IKA T25 Ultra Turrax*^®^ equipped with the dispersing element type *S25 N18G*.

The chemical compositions of most water dispersions used in the dip-coating process are given in the Table 1, and most often, they were as follows:
CuSiO_3_∙xH_2_O1–7 wt.%*CellosizeHEC QP-40*0.9–1.0 wt.%*Polikol 400* (PEG400)5–10 wt.%Dispersion of chosen acrylic resin (or PVAc)3–10 wt.%2 wt.% Water solution of soluble starch (or PVA) 25 wt.%Nanosilica (*Aerosil 380*)0.1–0.2 wt.%Silicone oil (*Polastosil M200*)0.1 wt.%Other additivesChanging amounts

Weighed samples of the nonwovens or fabrics were dip-coated in a horizontal two-shaft pad machine, with a roller pressure of 30 kG/cm^2^, in order to obtain the appropriate degree of application. Next, the textile materials were dried in a *KTF-350S* coater-heating device (Mathis, Niederhasli, Switzerland) at a temperature of 100 °C for 3 min, followed by heating at 140 °C for the next 1 min.

#### 2.3.3. Description of the Coating Method for Polymer Fabrics and Nonwovens 

For the fabric-coating process, aqueous pastes containing the following ingredients in appropriate amounts were prepared most often:
3–10 wt.% Reagents with antimicrobial properties (e.g., Cu silicate or/and metal oxide)5–10 wt.%Wetting agent3–10 wt.%Binders and thickeners

Coating pastes were applied to the surface of selected fabrics or nonwoven fabrics using a knife supported over a roller, using a laboratory kit for applying nanostructured coatings, KTF-350S (Mathis, Switzerland). Coatings with a thickness of 0.1 mm were applied. The samples were dried and reheated under the same conditions as after the coating process. The degree of application of the so-called dry weight of hybrid modifiers and the surface weight of modified textile materials depended on the chemical composition of the dispersions used and on the type of fabric or nonwoven. The degree of dry substance application (*SNS*) was calculated with the formula:

$$SNS=\frac{m_{p}-m_{s}}{m_{s}}\times 100\;[\%]$$

where: 

*m_s_*—weight of the textile sample before coating (g),

*m_p_*—weight of the textile material sample after coating, drying, and heating (g).

In addition, the surface mass of each modified nonwoven or fabric sample (g/m^2^) was determined.

#### 2.3.4. Description of Dip-Coating and Coating Experiments (Chosen Examples)

The method of antimicrobial modification of nonwovens and fabrics is illustrated with the following examples, described in detail.

**Example 1.**  *A polyester nonwoven fabric (aqua-jet Hydronina^®^) with a surface mass of 100 g/m^2^ and dimensions of 34 cm × 55 cm × 0.07 cm was dip-coated in a roller pad machine (roller pressure of 30 kG/cm^2^) with a water dispersion containing 6.0 wt.% copper silicate hydrate, 1.0 wt.% HEC, and 10.0 wt.% PEG400. After dip-coating, a weighed sample of the nonwoven fabric was dried at 100 °C for 3 min and heated at 140 °C for 1 min in the Mathis KTF-350S device. The modified sample of the nonwoven fabric, cooled down to room temperature for 1 h, was weighed again, the degree of modifier deposition (26.0% by weight) was calculated, and its antimicrobial properties were analyzed. The tested sample showed an antibacterial activity coefficient of A = 6.2, a bacteriostatic coefficient of S = 6.7, a bactericidal coefficient of L = 2.0, and a reduction in the colony growth of Gram-negative bacteria Escherichia coli (ATCC 25922), R = 96.8%. It also showed a coefficient of antibacterial activity A = 5.2, bacteriostatic coefficient S = 5.4, bactericidal coefficient L = 2.0, and a reduction in the colony growth of Gram-positive bacteria Staphylococcus aureus (ATCC 6538), R = 89.6%.*

**Example 2.**  *A polyester nonwoven fabric (aqua-jet Hydronina) with a surface mass of 100 g/m^2^ and dimensions of 34 cm × 55 cm × 0.07 cm was dip-coated in a roller pad machine (roller pressure of 30 kG/cm^2^) with a water dispersion containing 6.0 wt.% composite oxide hydrate ZnO·SiO_2_·xH_2_O [45], 1.0 wt.% HEC, 10.0 wt.% PEG400, and 10 wt.% Revacryl 247^®^ styrene–acrylic resin. After padding, a weighed sample of this nonwoven fabric was dried at 100 °C for 3 min and annealed at 140 °C for 1 min in the Mathis KTF-350S device. The modified sample of the nonwoven fabric, cooled down to room temperature (RT) for 1 h, was weighed again, the degree of modifier deposition was calculated (28.4% by weight), and its antimicrobial properties were analyzed. The tested sample showed an antibacterial activity coefficient of A = 6.0, a bacteriostatic coefficient of S = 6.6, a bactericidal coefficient of L = 2.1, and a reduction in the colony growth of Gram-negative bacteria Escherichia coli (ATCC 25922), R = 97.0%. It also showed an antibacterial activity coefficient of A = 4.8, bacteriostatic coefficient S = 4.9, bactericidal coefficient L = 0.6, and a reduction in the colony growth of Gram-positive Staphylococcus aureus (ATCC 6538), R = 70.8%.*

**Example 3.**  *A polyester twill fabric with a surface mass of 140 g/m^2^ and a wetting angle of 112.6° was subjected to enzymatic treatment at 35 °C for 30 min in a bath containing 1, 1.5, or 2 wt.% Texazym PES^®^ esterase enzyme at pH 4.2 (adjusted with the addition of acetic acid). The bath ratio was 10:1. The enzymatically modified samples of the PES fabric were dried at 100 °C for 2 min. After enzymatic modification, the wetting angle between the PES fabric surface and water decreased to 82.9°, 66.9°, and 35.9° for the enzyme content in the bath of 1, 1.5, and 2 wt.%, respectively. A PES fabric sample with dimensions of 34 cm × 55 cm × 0.04 cm was modified with 2 wt.% Texazym PES^®^ enzyme, coated with a paste containing 10.21 wt.% copper silicate hydrate (CuSiO_3_·xH_2_O), 5.11 wt.% PEG600, 10.21 wt.% Revacryl 247^®^, and 1.72 wt.% synthetic acrylic thickener Lutexal Thickener HC^®^. The coating paste was applied to the surface of the fabric using the supported knife technique (knife over a roller), using a laboratory device for applying nano-structured coatings, Mathis KTF-350S. The thickness of the coating was adjusted by adjusting the feeding gap with a feeler gauge. The width of the gap between the knife and the roller used was 0.1 mm. Immediately after the coating process, a sample of the PES fabric was dried and heated at 160 °C for 2 min in the Mathis KTF-350S apparatus. The tested sample showed an antibacterial activity coefficient of A = 5.5, a bacteriostatic coefficient of S = 5.4, a bactericidal coefficient of L = 2.2, and a reduction in the growth of the Candida albicans yeast fungus (ATCC 10231), R = 99.5%.*

**Example 4.**  *A The PES FS F-5 nonwoven fabric with a surface mass of approx. 240 g/m^2^ was alkalized for 4 days at room temperature in an aqueous solution with a concentration of 2 wt.% NaOH and 4 wt.% Na_2_CO_3._ Next, it was pressed in a roller wringing machine, immersed in demineralized water, and again pressed in the wringing machine. After drying in the KTF-350S apparatus (Mathis), at a temperature of 120 °C for 4 min and at 140 °C for 2 min, a weighed sample of this PES nonwoven fabric (with dimensions of 34 cm × 55 cm × 0.8 cm) was dip-coated in a roller pad machine (at a roller pressure of 30 kG/cm^2^) with water dispersion no. 14 (according to the data in Table 1), previously mixed for 2 min with a mechanical paddle stirrer at a speed of 2000 rpm, containing 7.0 wt.% copper silicate hydrate and other components of the mixture. After dip-coating, a weighed sample of the PES FS F-5 nonwoven fabric was dried at 120 °C for 4 min and heated at 140 °C for 2 min in the Mathis KTF-350S device. The modified PES FS F-5 samples, cooled for 1 h, were weighed again, their surface mass was determined (402.3 g/m^2^), the degree of modifier deposition was calculated (25.6 wt.%), and their antimicrobial properties were tested. The tested samples showed a reduction in the colony growth of Gram-negative Escherichia coli (ATCC 25922) (R > 99.98%), a reduction in the growth of Gram-positive bacteria Staphylococcus aureus (ATCC 6538) (R = 99.5%,) and a reduction in the growth of the Candida albicans yeast fungus (ATCC 10231) (R = 49.5%).*

**Example 5.**  *A PES WIFP-270 nonwoven fabric with a surface mass of approx. 270 g/m^2^ was alkalized for 4 days at room temperature in an aqueous solution with a concentration of 2 wt.% NaOH and 4 wt.% Na_2_CO_3_, pressed in a roller wringing machine, immersed in demineralized water, and again pressed in the wringing machine. After drying in the Mathis KTF-350S apparatus (at 120 °C for 4 min and at 140 °C for 2 min), a weighed sample of this PES nonwoven fabric with dimensions of 34 cm × 55 cm × 0.35 cm was dip-coated in a roller pad machine (with a roller pressure of 30 kG/cm^2^) with water dispersion no. 16 (according to the data in Table 1), containing 5 wt.% copper silicate hydrate and 2.1 wt.% TiO_2_ and other components of the mixture. After dip-coating, the weighed sample of the PES WIFP-270 nonwoven fabric was dried at 120 °C for 4 min and heated at 140 °C for 2 min in the Mathis KTF-350S device. The modified sample of the PES WIFP-270 nonwoven fabric, cooled for 1 h to RT, was weighed again. Its surface mass was determined (394.2 g/m^2^), the degree of modifier deposition was calculated (31.2% by weight), and its antimicrobial properties were tested. The tested sample showed a reduction in the colony growth of Gram-negative bacteria Escherichia coli (ATCC 25922) (R > 99.97%), a reduction in the growth of Gram-positive bacteria Staphylococcus aureus (ATCC 6538) (R = 99.7%), and a reduction in the growth of Candida albicans (ATCC 10231) (R = 58.8%).*

**Example 6.**  *A PES WIFP-270 nonwoven fabric with a surface mass of approx. 270 g/m^2^ was alkalized for 4 days at room temperature in an aqueous solution with a concentration of 2 wt.% NaOH and 4 wt.% Na_2_CO_3_. Next, it was pressed in a roller wringing machine, immersed in demineralized water, and again pressed in the wringing machine. After drying in the Mathis KTF-350S device (at a temperature of 120 °C for 4 min and at 140 °C for 2 min), a weighed sample of the PES nonwoven fabric with dimensions of 34 cm × 55 cm × 0.35 cm was dip-coated in a roller pad machine (at a roller pressure of 30 kG/cm^2^) with water dispersion no. 17 (according to the data in Table 2), previously mixed for 2 min with a paddle mechanical stirrer at a speed of 2000 rpm, containing 5.0 wt.% copper silicate hydrate and 4.2 wt.% TiO_2_ and other components of the mixture. After padding, the weighed sample of the WIFP-270 nonwoven fabric was dried at 120 °C for 4 min and heated at 140 °C for 2 min in the Mathis KTF-350S apparatus. The modified sample of the PES WIFP-270 nonwoven fabric, cooled for 1 h to RT, was weighed again, its surface mass was determined (464.7 g/m^2^), the degree of modifier deposition was calculated (41.0% by weight), and the antimicrobial properties were tested. The tested sample showed a reduction in the colony growth of Gram-negative Escherichia coli (ATCC 25922) (R > 99.97%), a reduction in the colony growth of Gram-positive bacteria Staphylococcus aureus (ATCC 6538) (R = 99.5%), and a reduction in the growth of the Candida albicans yeast fungus (ATCC 10231) (R = 80.9%).*

#### 2.3.5. Evaluation of Antimicrobial Activity of Polymeric Nonwovens Modified with Aqueous Dispersions Containing Copper Silicate

The analysis of the biological activity of selected samples of functionalized polymer nonwovens was carried out in the Biodegradation and Microbiological Research Laboratory of our institute. The biological activity of the surface of biofunctionalized polymer nonwovens was analyzed in appropriate antibacterial tests against colonies of Gram-negative bacteria *Escherichia coli* (ATCC 11229) and Gram-positive bacteria *Staphylococcus aureus* (ATCC 6538) and in antifungal tests against the fungus *Candida albicans* (ATCC 10321)—according to the PN-EN ISO 20743:2021-12-02, Polish and European standard (Textiles—Determination of antibacterial activity of textiles).

#### 2.3.6. Scanning Electron Microscopy (SEM)

The surface morphology of polymeric nonwovens, before and after the biofunctionalization process, was analyzed using scanning electron microscopy (SEM). A microscopic analysis of fibers and fibrins, forming the nonwovens’ physical structures, was performed using a scanning electron microscope, model Quanta 200 (FEI Company, Singapore), equipped with a Q150R S vacuum sputtering machine. The SEM analysis was carried out in high vacuum, using a probe beam energy of 5 ekV. The magnification of the obtained SEM pictures was from 100 to 1000 times.

## 3. Research Results and Discussion 

The aim of this research was to develop stable water dispersions of various chemical compositions intended for the biofunctionalization of textile materials. For our study, we chose PES and PLA nonwovens with a surface mass of 210–350 g/m^2^, which should be suitable for future fabrication of polymeric nonwoven filters and their further application as filter cartridges for the apparatus used in air-conditioning systems. Other textile materials (cotton fabric, PES fabric, and PES nonwoven fabric *Hydronina*) chosen for our study are commonly applied in the textile industry, for instance, in the production of garments. In our research, dispersants from the group of synthetic acrylic and vinyl polymers, and a natural polysaccharide polymer (soluble starch) were used. These compositions formed homogeneous and stable water dispersions containing microorganism growth inhibitors out of a group of different hybrid modifiers:

- Copper silicate hydrate (CuSiO_3_∙18.5H_2_O) (which is a synthetic version of minerals: *chrysicolla* and *dioptase*),
- Titanium dioxide, zinc oxide, zinc silicate (ZnO∙SiO_2_), and zinc lactate,
- Their mixtures.

Such compositions were used for the antimicrobial biofunctionalization of textile materials, i.e., polyester (PES) and polylactide (PLA) nonwovens, as well as cotton, polyester, and cotton–polyester fabrics.

In our research, we used stable aqueous dispersions that contained 1–11 wt.% copper silicate hydrate, most preferably in the amount of at least 5–7 wt.% titanium dioxide in the form of micronized powder (*TK44*) or in the form of the commercial aqueous acrylic dispersion containing 20–22 wt.% TiO_2_ nanoparticles (*Cinkarna CCA 100BS*) and emulsion paints containing titanium dioxide (*Talens Amsterdam* and *Dekoral Silver*) and acrylic (*Titanium IN*) or silicate (*Titanium FA*) water dispersions with photocatalytic properties.

As dispersants and pro-adhesion agents, the above-mentioned stable dispersions contained diluted aqueous solutions of poly (vinyl alcohol) (PVA) or water-soluble starch in the amount of 2.0–5.0% by weight. As a thickening agent in these stable dispersions, hydroxyethyl cellulose (with the addition of sodium acetate) was used in the amount of 0.5–2.0% by weight, most often 0.8–1.5 wt.%. As a wetting agent, they contained liquid poly(ethylene glycol) (either *Polikol 400* or *Polikol 600*, or *Pluriol E600*) in the amount of 5–10% by weight, and as a plasticizer, they contained bis(2-ethylhexyl adipate) (*Adoflex*) in the amount of 25–50% by weight or/and glycerin in the amount of 0.4–1.0 wt.% (most often 0.5 wt.%) — with respect to the amount of water dispersion of poly(vinyl acetate) (*Synexil DN-50*) used.

A hydroxyl-terminated methyl silicone oil (*Polastosil M200*) was used as an antifoaming agent in the dispersions, in the amount of 0.1–0.25% by weight, most often 0.1 wt.%.

### 3.1. An Improvement in the Surface Wettability and Hydrophilicity of Textile Materials

In order to improve the wettability of the surface, enzymatic treatment of nonwovens and fabrics was carried out at a temperature of 30–35 °C in an aqueous solution containing 1–2 wt.% enzyme *Texazym PES* (from the group of esterases) at pH 4–4.5. The ratio of bath volume to textile weight (the so-called *bath ratio*) was 10:1. Alkaline treatment of the tested PES nonwovens was carried out at room temperature by immersing them in a solution of 2–6 wt.% NaOH and Na_2_CO_3_ for 3–4 days, and then, the nonwoven fabric was squeezed to remove excess alkali solution, rinsed in water, squeezed again, and dried and heated at 120–140 °C.

However, the alkaline treatment of fabrics was carried out at a temperature of 95–98 °C, for 60 min in a bath containing sodium hydroxide at a concentration of 1.8 g/L, sodium carbonate at a concentration of 3.6 g/L, and a sequestering and wetting agent. The ratio of bath volume to textile weight used was also 10:1.

The effectiveness of the pretreatment of polyester textile carriers was evaluated by determining the contact angle and the surface free energy (SFE) using the goniometric method. As the result of the modification of the polyester fabric (PES) surface with the use of an enzyme from the group of esterases, *Texazym PES* (1–2 wt.%), the hydrophilicity of PES fibers was improved, resulting in a decrease in the contact angle (against water) from 112.6° for the initial PES fabric to 82.9°, 66.9°, and 35.9° for the PES fabric after modification with *Texazym PES* enzyme used at concentrations of 1.0, 1.5, and 2.0 wt.%, respectively. This was accompanied by an increase in the SFE from 50.9 mJ/m^2^ for the original PES fabric to values of 51.9, 56.9, and 59.5 mJ/m^2^, respectively. The results of these tests are given in Table 2.

However, as a result of the alkaline treatment of the polyester fabric, the contact angle, measured with a water drop, decreased from 112.6° for the initial PES fabric to 103.2° and the SFE increased from 50.9 mJ/m^2^ for the initial PES fabric to 52.8 mJ/m^2^ after alkalization.

The stable water dispersions described here were used for the biofunctionalization of polyester or biodegradable PLA nonwovens (with a surface mass of 100–350 g/m^2^) using the dip-coating method for the biofunctionalization of cotton, polyester, and cotton–polyester fabrics with a surface mass of 100–170 g/m^2^, which were initially subjected to enzymatic or alkaline treatment. The initial surface modification improved the wettability and adhesive properties of hydrophobic polymer fabrics and nonwovens (PES and PLA).

### 3.2. Evaluation of the Results of Antimicrobial Modification of the Properties of Nonwovens and Fabrics Modified with Aqueous Dispersions Containing Copper Silicate

The biological activity of the surface of biofunctionalized polymer nonwovens was analyzed in appropriate antibacterial tests against colonies of Gram-negative *Escherichia coli* (ATCC 11229) and Gram-positive *Staphylococcus aureus* (ATCC 6538) and in antifungal tests against *Candida albicans* (ATCC 10321)—according to the PN-EN ISO 20743 standard. The results of the analysis of the biological activity of selected samples of the functionalized polymer nonwovens are summarized in Table 3.

The polymeric PES and PLA nonwovens, modified on the surface with compositions containing copper silicate hydrate, showed good antibacterial properties against Gram-negative bacteria *Escherichia coli*, even at a content of 1 wt.% CuSiO_3_∙xH_2_O in the aqueous dispersions, and against Gram-positive bacteria *Staphylococcus aureus*, at a content of at least 5 wt.% CuSiO_3_∙xH_2_O in the aqueous dispersions. The bacterial growth reduction factor (R) was greater than 99% for most of the samples tested. Values of the factor R (%)**,** determined against different microorganisms, correspond to the content of killed bacterial cells in contact with the applied antimicrobial agents.

Good antifungal properties against the fungus *Candida albicans* were found for the PES and PLA nonwoven fabrics modified with dispersions containing 5–7 wt.% CuSiO_3_∙xH_2_O and 4.2–5.0 wt.% TiO_2_. The addition of TiO_2_ caused a significant improvement in the antifungal properties of PES and PLA nonwovens modified in this way. For the samples of PES *WIFP-270* and *FS F-5* nonwovens modified with water dispersions containing 5.0 wt.% CuSiO_3_∙xH_2_O and 4.2–5.0 wt.% TiO_2_ (and alternatively also with the addition of other commercial dispersions containing TiO_2_), the growth reduction factor for the fungus *Candida albicans* (R) reached values in the range of 80.9–98.0% (see the data in Table 3).

#### Scanning Electron Microscopy (SEM) of Nonwoven Samples

**Figure 1:**
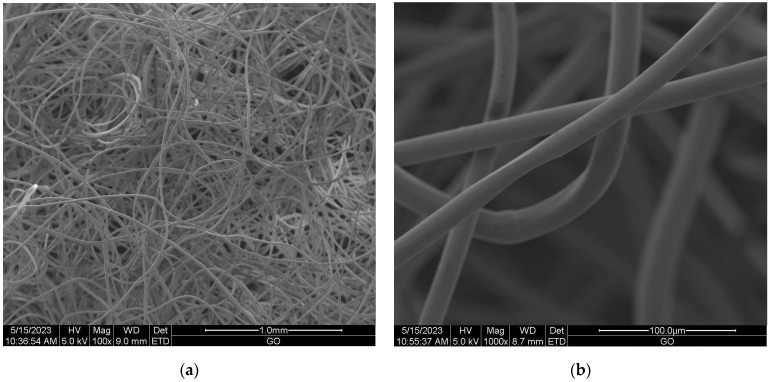
The SEM images of unmodified PES nonwoven *WIFP-270*; magnification: ×100 (**a**) and ×1000 (**b**).

**Figure 2:**
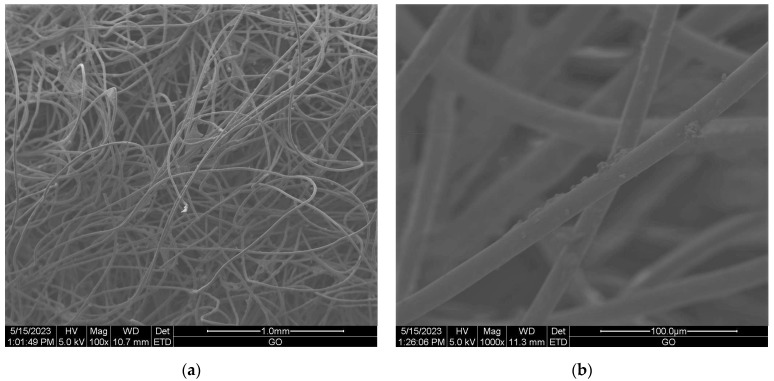
The SEM images of PES nonwoven *WIFP-270* (from exp. no. 20 in Table 1 and no. 16 in Table 2) modified with 5.0 wt.% CuSiO_3_ and 5.0 wt.% TiO_2_; magnification: ×100 (**a**) and ×1000 (**b**).

**Figure 3:**
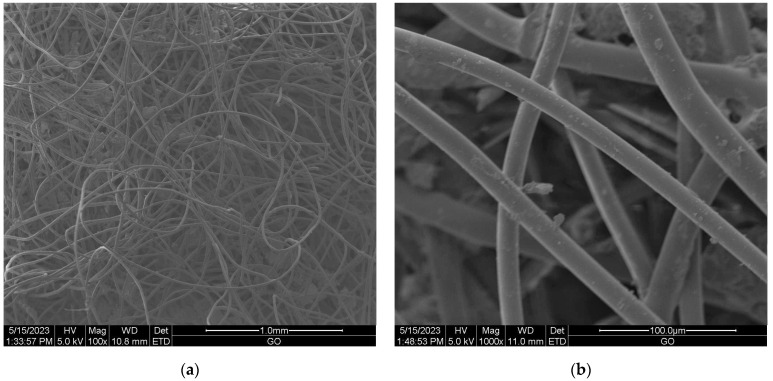
The SEM images of PES nonwoven *WIFP-270* (from exp. no. 21 in Table 1 and no. 19 in Table 2), modified with 5.0 wt.% CuSiO_3_ + 5.0 wt.% TiO_2_ + 2 wt.% *Dekoral Silver*; magnification: ×100 (**a**) and ×400 (**b**).

**Figure 4:**
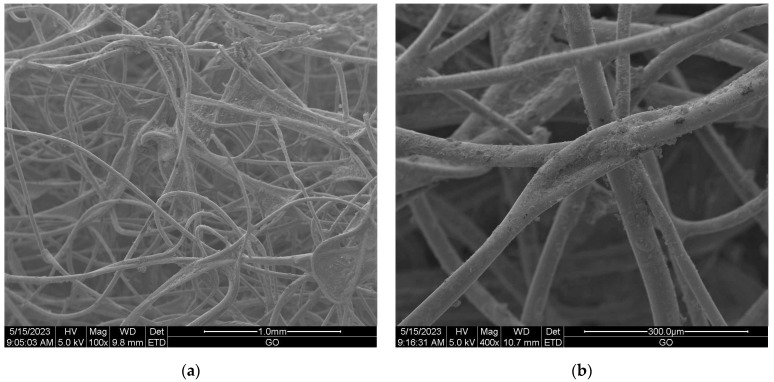
The SEM images of PES nonwoven *WIFP-270* (from exp. no. 23 in Table 1 and no. 20 in Table 2) modified with 5.0 wt.% CuSiO_3_ and 25.0 wt.% *Titanium IN*; magnification: ×100 (**a**) and ×400 (**b**).

**Figure 5:**
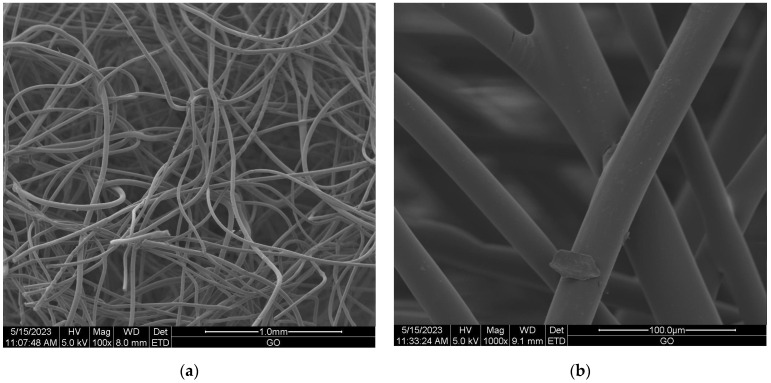
SEM images of unmodified PES nonwoven *FS F-5*; magnification: ×100 (**a**) and ×1000 (**b**).

**Figure 6:**
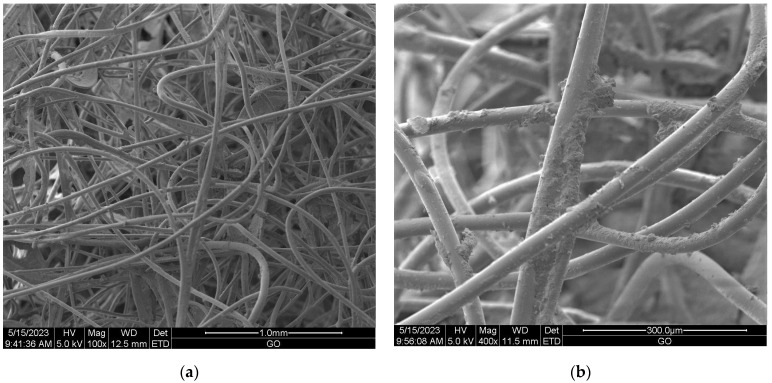
The SEM images of PES nonwoven *FS F-5* (from exp. no. 20 in Table 1 and no. 17 in Table 2) modified with 5.0 wt.% CuSiO_3_ and 5.0 wt.% TiO_2_; magnification: ×100 (**a**) and ×400 (**b**).

**Figure 7:**
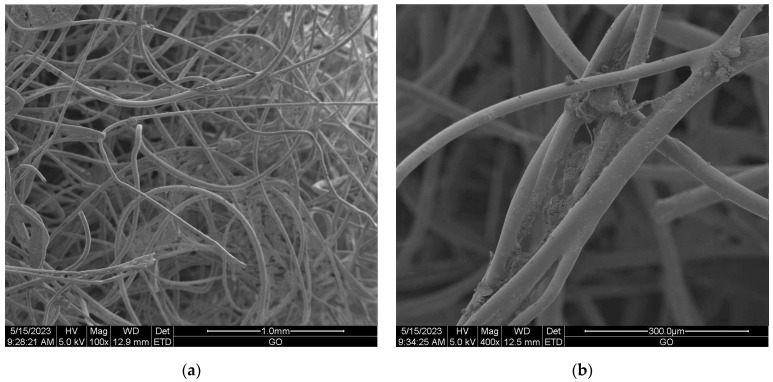
The SEM images of PES nonwoven *FS F-5* (from exp. no. 21 in Table 1 and no. 18 in Table 2) modified with 5.0 wt.% CuSiO_3_ + 5.0 wt.% TiO_2_ + 2 wt.% *Dekoral Silver*; magnification: ×100 (**a**) and ×400 (**b**).

**Figure 8:**
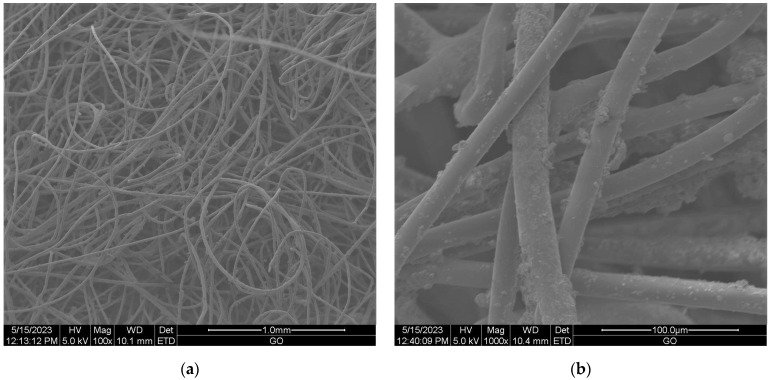
The SEM images of PES nonwoven *FS F-5* (from exp. no. 23 in Table 1 and no. 21 in Table 2) modified with 5.0 wt.% CuSiO_3_ and 5.0 wt.% *Titanium IN*; magnification: ×100 (**a**) and ×1000 (**b**).

Scanning electron microscopy (SEM) provides imaging of surfaces or cross sections of various types of materials, enabling their testing and analysis. In Figure 1, Figure 2, Figure 3, Figure 4, Figure 5, Figure 6, Figure 7 and Figure 8 are presented SEM pictures of the starting polymeric nonwoven fabrics, used in our study, and the obtained biofunctionalized nonwoven samples—after dip-coating and drying processes. In these pictures, it can be seen that microparticles of coating compositions adhered well to the surfaces of the fibers, which formed a network of PES nonwovens.

### 3.3. Evaluation of the Results of the Antimicrobial Modification of the Properties of Fabrics (and Nonwovens) with Copper Silicate and Composite Hybrid Oxide ZnO∙SiO_2_ Using the Dip-Coating and Coating Methods 

The results of the research conducted on the antimicrobial modification of fabrics and one PES nonwoven fabric are presented only in the form of a summary. The surfaces of these textile materials were first modified with 2.0 wt.% *Texazym PES* before the biofunctionalization process—as described in Section 3.1.

This part of our study showed that:

1. The cotton fabric *Medical*, the polyester fabric, the cotton–polyester fabric *Figaro*, and also the polyester nonwoven fabric *Hydronina*, biofunctionalized with the dip-coating method with dispersions containing 6.0 wt.% CuSiO_3_∙xH_2_O (where: x = 18.5) or the composite hybrid oxide ZnO∙SiO_2_ exhibited:
  (a) Against the Gram-negative bacteria *Escherichia coli*
    - Strong antibacterial properties (the antibacterial activity coefficient A reached values in the range of 3.1–6.2 for the samples modified with CuSiO_3_∙xH_2_O and 3.7–6.0 for the samples modified with ZnO∙SiO_2_),
    - Strong and significant bacteriostatic properties (the bacteriostatic coefficient S reached values in the range of 3.0–6.7 for the samples modified with CuSiO_3_∙xH_2_O and 2.1–6.6 for the samples modified with ZnO∙SiO_2_),
    - Good bactericidal properties (the bacterial growth reduction factor R was 77.5–96.8% for the samples modified with CuSiO_3_∙xH_2_O and 72.3–97.0% for samples modified with ZnO∙SiO_2_).
  (b) Against Gram-positive bacteria *Staphylococcus aureus*
    - Strong or significant antibacterial properties (the antibacterial activity coefficient A reached values in the range of 2.5–6.2 for the samples modified with CuSiO_3_∙xH_2_O and 2.8–4.8 for the samples modified with ZnO∙SiO_2_),
    - Strong and significant bacteriostatic properties (the bacteriostatic coefficient S reached values in the range of 2.6–6.5 for the samples modified with CuSiO_3_∙xH_2_O and 3.0–5.2 for the samples modified with ZnO∙SiO_2_),
    - Good and significant bactericidal properties (the bacterial growth reduction factor R was 89.6–99.0% for the samples modified with CuSiO_3_∙xH_2_O and 70.8% for the samples modified with ZnO∙SiO_2_).
2. For all samples of the PES fabric and the PES nonwoven *Hydronina*, modified with the coating method with a paste containing approx. 10 wt.% CuSiO_3_∙xH_2_O, the growth reduction factor for *Candida albicans* (R) reached values in the range of 97.9–99.6%, the antibacterial activity coefficient A was in the range of 4.8–5.6, the bacteriostatic coefficient S had the same value 4.8–5.6, and the bactericidal coefficient L was in the range of 1.6–2.4.
3. The obtained composite polymer textile materials also showed a good inhibitory effect on the development of the mold fungus *Chaetomium globosum*. The samples of the textile materials coated with CuSiO_3_ hydrate, and especially the polyester fabric subjected to biomodification with 7.0 wt.% CuSiO_3_∙xH_2_O, showed a clear effect of antifungal activity against the fungus *Chaetomium globosum*, which grew on the surface of the samples only in the range of 0–25%.
4. The new biofunctionalized textile materials obtained using the coating method (mainly cotton, cotton–polyester, and polyester fabrics) with pastes containing (a) CuSiO_3_ or (b) CuSiO_3_ + ZnO, or (c) CuSiO_3_ + TiO_2_ particles introduced onto the surface and incorporated into their structures also showed good barrier properties against UV radiation (UPF > 50) and the lowest transmittance (T average was 2.5–3.5), which was characteristic of the textile products subjected to the initial alkaline or biochemical (enzymatic) modifications, followed by biofunctionalization with mixtures containing a total of 10 wt.% CuSiO_3_∙xH_2_O and TiO_2_ (or ZnO) in a weight ratio of 7:3 or 1:1.

**Figure 9:**
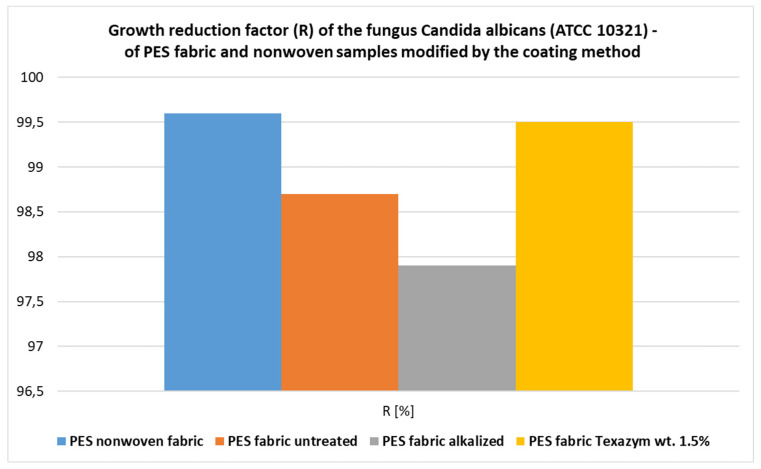
The values of the growth reduction factor for the fungus *Candida albicans* (R), determined for different textile materials that were biofunctionalized with copper silicate hydrate using the coating method.

**Figure 10:**
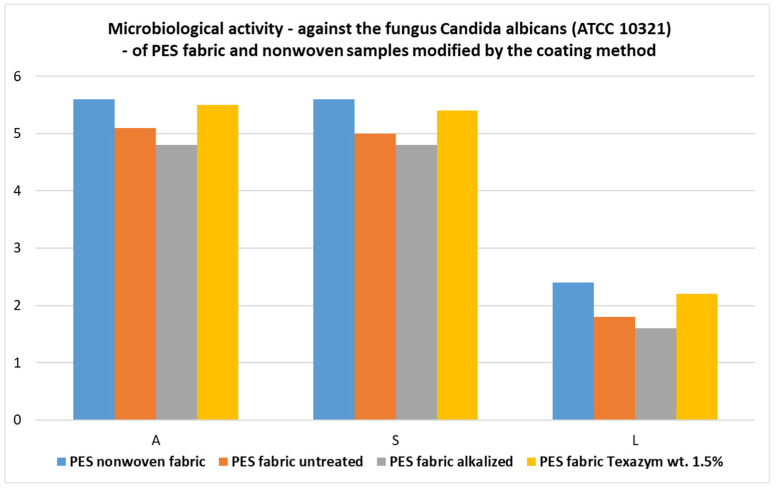
The values of the antibacterial activity coefficient **A**, the bacteriostatic coefficient **S**, and the bactericidal coefficient **L**, determined against the fungus *Candida albicans*, for different textile materials functionalized with copper silicate hydrate using the coating method.

The results of the microbiological tests of the PES fabrics and the PES nonwoven *Hydronina* modified with the coating method with pastes containing approx. 10 wt.% CuSiO_3_∙xH_2_O are listed in Table 4 and presented in graphical form in Figure 9 and Figure 10.

Evaluation criteria according to EN ISO 20743 (2021) standard
Evaluation of antimicrobial activityReduction in microbial growth **(A)**
Lack**A** < 0.5Weak0.5 ≤ **A** < 2Significant2 ≤ **A** < 3Strong**A** ≥ 3

## 4. Conclusions

PES and PLA polymer nonwovens, modified on the surface with stable water compositions containing copper silicate hydrate, showed good antibacterial properties against the Gram-negative bacteria *Escherichia coli*, even at a content of 1 wt.% CuSiO_3_∙xH_2_O in the aqueous dispersions, and against the Gram-positive bacteria *Staphylococcus aureus* at a content of at least 5 wt.% CuSiO_3_∙xH_2_O in the aqueous dispersions. The bacterial growth reduction factor (R) was greater than 99% for most of the samples tested.

Moreover, good antifungal properties against the fungus *Candida albicans* were found for the PES and PLA nonwoven fabrics modified with dispersions containing 5–7 wt.% CuSiO_3_∙xH_2_O and 4.2–5.0 wt.% TiO_2_. The addition of TiO_2_ caused a significant improvement in the antifungal properties of the PES and PLA nonwovens modified in this way. For the samples of PES *WIFP-270* and *FS F-5* nonwovens modified with water dispersions containing 5.0 wt.% CuSiO_3_∙xH_2_O and 4.2–5.0 wt.% TiO_2_ (and possibly with the addition of other dispersions containing TiO_2_), the growth reduction factor for the fungus *Candida albicans* (R) reached values in the range of 80.9–98.0% (see Table 3).

Polymer nonwovens biofunctionalized with water dispersions containing CuSiO_3_ hydrate and TiO_2_ can be used in the production of filters for hospital air-conditioning systems and for the automotive industry, as well as in air purification devices. However, fabrics with antimicrobial properties, modified in a similar way using the dip-coating or coating methods (or with dispersions containing ZnO or hybrid zinc silicate ZnO∙SiO_2_), can be applied, for example, in the manufacture of mold-and fungi-resistant outdoor furniture.

## Figures and Tables

**Table 1:** Chemical compositions (in % by weight) of the selected water dispersions used in the process of dip-coating polymer nonwovens.

Components of Dispersions	Water Dispersion Number																			
	1	2	5	6	7	10	11	12	13	14	15	16	17	18	19	20	21	22	23	24
CuSiO_3_∙xH_2_O ^a^	3.0	5.0	3.0	2.0	1.0	1.0	3.0	5.0	5.0	7.0	6.9	7.0	5.0	5.0	7.0	5.0	5.0	7.0	5.0	5.0
* Cellosize * *HEC QP-40*	1.5	1.0	1.0	0.9	0.9	0.9	0.8	1.0	1.0	1.0	1.0	1.0	1.0	1.0	1.0	1.0	1.0	1.0	1.0	1.0
*Polikol 400* (PEG400)	7.5	5.0	-	-	-	-	6.0	7.5	-	-	10.0	10.0	7.5	7.5	-	7.5	7.5	10.0	10.0	10.0
*Pluriol E600* (PEG600)	-	-	7.5	5.0	5.0	5.0	-	-	7.5	10.0	-	-	-	-	10.0	-	-	-	-	-
Copolymer dispersion *Revacryl 247*	-	5.0	-	-	-	-	-	-	-	-	-	-	-	-	-	-	-	-	-	-
*Cinkarna 100 BS*	-	-	-	-	-	-	-	-	-	-	1.0 ^b^	2.1 ^b^	4.2 ^b^	-	-	5.0 ^b^	5.0 ^b^	-	-	-
*Talens Amsterdam Acrylic Binder 005* ^c^	4.5	-	3.0	5.0	3.0	5.0	3.0	1.0	1.0	1.0	-	-	-	-	1.0	-	-	-	-	-
Acrylic dispersion *Dekoral Silver* ^c^	-	-	-	-	-	-	-	-	-	-	-	-	-	-	-	2.0	2.0	-	-	-
Photocatalytic acrylic dispersion *Titanium IN* ^c^	-	-	-	-	-	-	-	-	-	-	-	-	-	-	-	-	-	-	25.0	-
Photocatalytic silicate dispersion *Titanium FA* ^c^	-	-	-	-	-	-	-	-	-	-	-	-	-	-	-	-	-	-	-	25.0
Zinc lactate pure	-	-	-	-	-	-	-	-	-	-	-	-	-	5.0	-	-	-	-	-	-
Glycerin	-	-	0.5	0.4	0.4	0.4	0.5	1.0	1.0	1.0	0.5	0.5	0.5	0.5	1.0	0.5	0.5	0.5	0.5	0.5
*Synexil DN-50*	-	-	-	-	-	-	1.0	4.0	4.0	4.0	2.50	2.0	2.0	2.0	4.0	2.0	-	-	-	-
Bis(2-ethylhexyl adipate) (*Adoflex*)	-	-	-	-	-	-	0.5	2.0	2.0	2.0	1.25	1.0	1.0	1.0	1.0	1.0	-	-	-	-
2 wt.% Water solution of soluble starch	-	-	25.0	25.0	25.0	25.0	25.0	25.0	25.0	25.0	25.0	25.0	25.0	25.0	25.0	25.0	25.0	25.0	25.0	25.0
5 wt.% Water solution of PVA	-	10.0	-	-	-	-	-	-	-	-	-	-	-	-	-	-	-	-	-	-
Nanosilica (*Aerosil 380*)	-	-	-	-	-	0.1	0.1	0.2	0.2	0.2	0.2	0.2	0.2	0.2	0.2	0.2	0.2	0.2	0.2	0.2
Silicone oil (*Polastosil M200*)	-	-	-	-	-	0.1	0.1	0.1	0.1	0.1	0.1	0.1	0.1	0.1	0.1	0.1	0.1	0.1	0.1	0.1
Demineralized water	83.5	74.0	60.0	61.5	64.5	62.5	60.0	53.2	53.2	48.7	48.5	43.2	37.7	51.7	49.7	33.9	34.9	31.2	33.2	33.2

^a^ Copper silicate hydrate (CuSiO_3_∙xH_2_O) with a water content of 18.5% by weight was used; ^b^ the numbers indicate the content of TiO_2_ in the water dispersion used in the dip-coating process; ^c^ in commercial aqueous dispersions (*Talens Amsterdam Acrylic Binder 005*, *Dekoral Silver*, *Titanium IN*, and *Titanium FA*), the content of TiO_2_ was not determined.

**Table 2:** Results of contact angle tests (Θ_W_, Θ_F_, and Θ_DIM_) for three standard liquids (water, formamide, and diiodomethane, respectively), the surface free energy (SFE) of polyester fabric samples, and sample images of water drops on the surfaces of tested polyester fabrics.

Wetted Surface	Image of a Drop of Water	Contact Angle Θ (deg)			Surface Free Energy (SFE) (mJ/m^2^)		
		Θ_W_	Θ_F_	Θ_DIM_	γ_S_^LW^	γ_S_^AB^	γ_S_
PES fabric (unmodified)	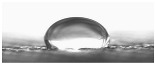	112.6	79.0	0.0	50.8	0.1	50.9
PES fabric + 1 wt.% *Texazym*	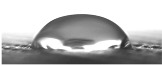	82.9	46.8	0.0	50.8	1.1	51.9
PES fabric + 1.5 wt.% *Texazym*	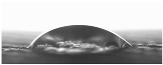	66.9	28.6	0.0	50.8	5.3	56.1
PES fabric + 2 wt.% *Texazym*	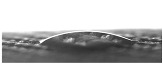	35.9	11.2	0.0	50.8	8.7	59.5
PES fabric after alkalization	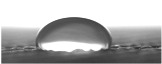	103.2	62.7	0.0	50.8	2.0	52.8

**Table 3:** The antibacterial and antifungal properties of the selected PES and PLA nonwovens modified by means of the dip-coating method with dispersions containing copper silicate hydrate (CuSiO_3_∙xH_2_O) *, titanium dioxide, and other components (% by weight)—according to the compositions given in Table 1.

No.	Type of Nonwoven Fabric Sample	Values of the Growth Reduction Factor (R) (%) against Different Microorganisms		
		*Escherichia coli* (ATCC 25922)	*Staphylococcus aureus* (ATCC 6538)	*Candida albicans* (ATCC 10231)
1.	PES WIFP-270 + 1.0% CuSiO_3_	89.7	43.0	0.0
2.	PES FS G-4 + 1.0% CuSiO_3_	97.4	16.8	0.0
3.	PES FS F-5 + 1.0% CuSiO_3_	99.1	20.6	1.4
4.	PLA-350 + 1.0% CuSiO_3_	>99.4	28.0	0.0
5.	PES WIFP-270 + 2.0% CuSiO_3_	99.6	59.1	26.8
6.	PES WIFP-270 + 3.0% CuSiO_3_	99.8	66.9	27.8
7.	PES WIFP-270 + 5.0% CuSiO_3_	>99.8	87.6	30.6
8.	PES FS F-5 + 5.0% CuSiO_3_	99.4	98.7	35.4
9.	PLA-350 + 5.0% CuSiO_3_	>99.8	97.0	30.8
10.	PES WIFP-270 + 7.0% CuSiO_3_	>99.98	94.4	37.6
11.	PES FS F-5 + 7.0% CuSiO_3_	>99.98	99.5	49.5
12.	PLA-350 + 7.0% CuSiO_3_	>99.98	>99.9	38.6
13.	PES WIFP-270 + 6.9% CuSiO_3_ + 1.0% TiO_2_	>99.97	92.2	48.5
14.	PES WIFP-270 + 7.0% CuSiO_3_ + 2.1% TiO_2_	>99.97	99.7	58.8
15.	PES WIFP-270 + 5.0% CuSiO_3_ + 4.2% TiO_2_	> 99.97	99.5	80.9
16.	PES WIFP-270 + 5.0% CuSiO_3_ + 5.0% TiO_2_	>99.99	99.4	87.5
17.	PES FS F-5 + 5.0% CuSiO_3_ + 5.0% TiO_2_	99.06	98.1	93.9
18.	PES FS F-5 + 5.0% CuSiO_3_ + 5.0% TiO_2_ + 2% *Dekoral Silver*	95.63	94.6	92.7
19.	PES WIFP-270 + 5.0% CuSiO_3_ + 5.0% TiO_2_ + 2% *Dekoral Silver*	97.08	92.6	98.0
20.	PES WIFP-270 + 5.0% CuSiO_3_ + 25% *Ti-IN*	>99.87	97.7	89.2
21.	PES FS F-5 + 5.0% CuSiO_3_ + 25% *Ti-IN*	>99.97	94.8	95.0

* Copper silicate hydrate (CuSiO_3_∙xH_2_O) with 18.5 wt.% content of H_2_O was used.

**Table 4:** The results of microbiological activity tests of PES fabric and nonwoven samples modified with the coating method with a paste containing about 10 wt.% CuSiO_3_∙xH_2_O containing PEG600 and the thickener agent *Lutexal HC*.

Sample No.	Sample Type	CuSiO_3_ xH_2_O	PEG600	*Revacryl 247*	*Lutexal HC*	Microbiological Activity against the Fungus *Candida albicans* (ATCC 10321)			
		(wt.%)				A	S	L	R (%)
1	PES nonwoven fabric (*Hydronina*)	10.36	5.18	10.36	1.67	5.6	5.6	2.4	99.6
2	PES fabric (untreated)	10.36	5.18	10.36	1.67	5.1	5.0	1.8	98.7
3	PES fabric alkalized	10.36	5.18	10.36	1.67	4.8	4.8	1.6	97.9
4	PES fabric + *Texazym* (1.5 wt.%)	10.21	5.11	10.21	1.72	5.5	5.4	2.2	99.5

Signs: **A**—the antibacterial activity coefficient; **S**—the bacteriostatic coefficient; **L**—the bactericidal coefficient; **R** (%)—the growth reduction factor of the fungus.

## Data Availability

The data are included in the text.
